# Kinase Mobility Shift Assay (KiMSA) for Assessing Protein Kinase A Activity

**DOI:** 10.21769/BioProtoc.5366

**Published:** 2025-07-05

**Authors:** Analia G. Novero, Tomás J. Steeman, Catalina Curcio, Lara Buccolini, Andres Binolfi, Diego Krapf, Mariano G. Buffone, Dario Krapf, Cintia Stival

**Affiliations:** 1 Cell Signal Transduction Networks Laboratory, IBR (CONICET-UNR), Rosario, Argentina; 2 Cellular-Structural Biology Laboratory, IBR (CONICET-UNR), Rosario, Argentina; 3 Department of Electrical and Computer Engineering, Colorado State University, Fort Collins, CO, USA; 4 Instituto de Biología y Medicina Experimental (CONICET), Ciudad Autónoma de Buenos Aires, Argentina

**Keywords:** Protein kinase A (PKA), Sperm capacitation, Kinase assay, cAMP, Fertility, Kemptide kinase activity, Non-radioactive assay, Phosphorylation, Gel electrophoresis, Fluorescence assay

## Abstract

The cAMP-dependent protein kinase (PKA) is one of the most extensively distributed kinases among intracellular signal cascades, with a pivotal role in the regulation of various processes, including the capacitation of sperm cells. Traditional assessments of PKA activity rely on the utilization of [γ-^32^P] ATP and the Kemptide peptide as a substrate. This strategy presents several major drawbacks, including high costs and health risks derived from the manipulation of radioactive isotopes. In this work, we introduce an enhanced non-radioactive assay to quantify PKA activity, termed kinase mobility shift assay (KiMSA), based on the use of a fluorescent-labeled Kemptide (Kemptide-FITC). Once the kinase reaction is terminated, the products can be easily resolved through electrophoresis on an agarose gel and quantified by fluorescence densitometry. We show that KiMSA is suitable for isolated PKA as well as for the enzyme in cell extracts. In addition, it enables quantification of PKA activity during the progression of mouse sperm capacitation. Furthermore, the assay enables monitoring the inhibition of PKA with pharmacological inhibitors in live cells. Therefore, the experimental and optimal assay conditions are set so that KiMSA can be used to assess in vitro as well as in vivo PKA activity in sperm cells. Finally, this method allows for measurement of cAMP concentrations, rendering a versatile technique for the study of cAMP/PKA pathways.

Key features

• KiMSA is a versatile kinase mobility shift assay for measuring PKA activity in sperm physiology, replacing radioactive assays with a fluorescence-labeled substrate for high sensitivity.

• This in vitro assay enables evaluation of activation state, drug effects, or PKA kinetics after purification or mutation.

• This assay measures PKA activity from cell extracts, reflecting pre-lysis activation status and intracellular signaling, though not a true in vivo readout.

• The standard protocol completion time is one day, including sperm preparation, kinase reactions, electrophoresis, and fluorescence quantification.

## Graphical overview


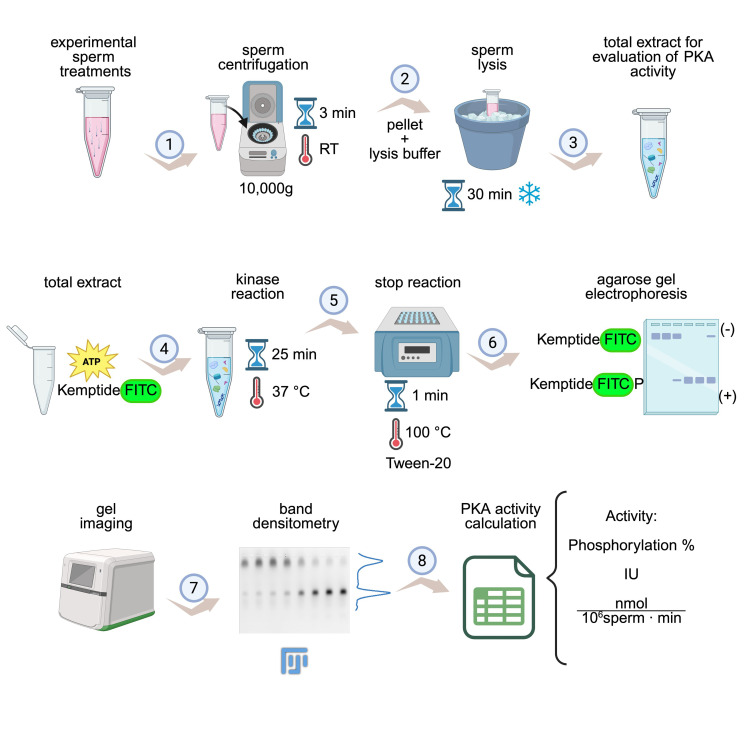


**KiMSA workflow summary.** Diagram illustrating the steps for obtaining cell extracts for assessing protein kinase A (PKA) activity. Sperm cells are first incubated under the desired experimental conditions (e.g., *non-capacitating* and *capacitating* treatments) and then centrifuged at room temperature (RT) for 3 min at 10,000× *g* (1). Lysis buffer is added to the pellet and incubated for 30 min on ice (2). The required total extract is ready for use (3). Kinase assay is carried out using total extract along with kinase buffer, for 25 min at 37 °C and in the dark (4). Reactions are stopped by quickly placing in ice, adding Tween-20, and incubating at 100 °C for 1 min (5), followed by running on agarose gel electrophoresis (6), gel imaging, and densitometry of phosphorylated and non-phosphorylated Kemptide-FITC signals (7). Finally, PKA activity is calculated as phosphorylation percentage or IU/normalized activity (8). Image modified from the original by Novero et al. [1].

## Background

For mammalian sperm to fertilize an oocyte, they first need to undergo a process known as sperm capacitation [2,3], which can be achieved both in vivo during their transit through the female reproductive tract or in vitro by incubating the sperm in a defined capacitating medium [4]. One of the first events during capacitation is the activation of the protein kinase A (PKA), also known as cAMP-dependent kinase A, which phosphorylates proteins on either Ser or Thr residues within the consensus phosphorylation sequence RRXS/T [5]. Early activation of PKA in sperm cells regulates several downstream signaling cascades involved in sperm capacitation [4,6]. In addition, a wide body of literature, which was initiated by the pioneer work of Walsh on phosphorylation related to cAMP-dependent protein kinase activity, indicates that PKA is responsible for phosphorylating a broad array of targets and is considered an essential regulator of many signaling events in somatic cells [7,8]. The PKA holoenzyme is constituted by two regulatory subunits (PKA-R) bound to two catalytic subunits (PKA-C), keeping the enzyme in an inactive state [9–11]. Sperm capacitation can be achieved in vitro upon incubation of sperm in media containing bicarbonate, among other culture medium components, named capacitating media. Bicarbonate stimulates the sperm soluble adenylate cyclase (sAC) [12], triggering an increase in intracellular cAMP. When sperm are incubated under capacitating conditions, the increase of intracellular cAMP induces a conformational change in the regulatory subunits of PKA that releases active PKA-C [5,10]. Since PKA is a central regulator of capacitation and, in turn, of male fertility, investigating its activation is highly relevant in the reproductive field. At present, the predominant approach for assessing direct PKA activity takes advantage of a specific peptide of eight amino acids (LRRASLGK) containing a consensus phosphorylation site for PKA named Kemptide [13,14]. By conducting a catalytic reaction in the presence of [γ-^32^P]-ATP, the amount of incorporated radioactive phosphate into the Kemptide can be easily correlated to PKA activity [15–17]. However, this methodology has several disadvantages related to the use of ^32^P, including health risks, high costs, the short half-life of the radioisotope, and the need for trained personnel with specialized facilities. To overcome these drawbacks, we developed a new methodology named KiMSA (Kinase Mobility Shift Assay), where we have labeled the Kemptide using FITC [LRRASLGK-(FITC)], resulting in a compound with a net negative charge at basic pH, which allows it to migrate toward the positive pole (anode) when subjected to agarose gel electrophoresis. Phosphorylation of Kemptide by PKA adds two negative charges to the peptide, resulting in increased migration toward the anode during electrophoresis, allowing the separation of the non-phosphorylated from the phosphorylated version [14,18]. This approach yielded similar results to the standard assay employing [γ^32^P]-ATP [19] and has been later applied to assess the in vitro activity of PKA purified from pig heart [14]. However, beyond comparable results, KiMSA enables real-time and high-throughput analysis, offering greater sensitivity and quantitative precision across a broader dynamic range. This method also reduces assay variability by directly monitoring substrate binding and phosphorylation events without the need for separation steps, making it more amenable to automation and large-scale screening applications. Collectively, these features position KiMSA as a more efficient, scalable, and safer option for assessing PKA activity in both basic research and drug discovery contexts. In the reproductive field, KiMSA can be easily adapted to measure PKA activity in sperm extracts, a key enzyme during the capacitation process. While previous attempts to use fluorescent-based methods were optimized for purified kinases [18], several issues arise when complex biological samples, such as sperm lysates, are used as the source of the kinase. The assay performance is then hampered by high background fluorescence, peptide aggregation, and debris, which obscure band resolution and reduce sensitivity. The comprehensive protocol provided herein can be used for extracting PKA under conditions that preserve its physiological activation state. Therefore, KiMSA advances the original concept by enabling robust quantification of PKA activity directly in cell extracts. Furthermore, KiMSA accurately reported PKA activation in both non-capacitated and capacitated sperm, truly reflecting the enzyme’s physiological state.

## Materials and reagents

**Biological materials**

1. Sperm from sexually mature C57BL/6 male mice (10–15 weeks old)

**Reagents**

*Note: Unless specified, all reagents are stored at room temperature (RT, 20–25 °C).*

1. Bovine serum albumin (BSA) fatty acid-free (Sigma-Aldrich, catalog number: A7906) (4 °C)

2. Adenosine 3-phosphate (ATP) (Sigma-Aldrich, catalog number: A7699) (-20 °C)

3. Cyclic AMP (cAMP) (Sigma-Aldrich, catalog number: A9501) (-20 °C)

4. PhosSTOP phosphatase inhibitor (Roche, catalog number: 4906837001) (4 °C)

5. cOmplete EDTA-free protease inhibitor cocktail (Roche, catalog number: 4693132001) (4 °C)

6. Non-fluorescent Kemptide (sequence LRRASLG) (AnaSpec, catalog number: 22594) (-80 °C)

7. Carboxyl-labeled fluorescent Kemptide (sequence LRRASLGK-FITC, termed Kemptide-FITC) (-80 °C) (Biomatik, catalog number: 1036600)

*Note: Additional information on Kemptide-FITC is as follows:*

*Origin: Synthesized by Biomatik. Sequence: N-LRRASLGK-C-(FITC). MW: 1,289.49 Da. Salt form: trifluoroacetate (TFA Salt). Solubility in water: 1 mg/mL. Purity (HPLC): 96.01%. FITC = fluorescein isothiocyanate. Weigh 1 mg of powder and dissolve it in 1 mL of water (do not weigh less than the resolution limit of the scale). Always keep in the dark to avoid quenching of FITC.*

8. Tris(hydroxymethyl)aminomethane (Tris base) (Cicarelli, catalog number: 1131214)

9. Triton X-100 (Neo Lab, catalog number: 01685)

10. NaCl (Cicarelli, catalog number: 750)

11. MgCl_2_ (Sigma-Aldrich, catalog number: M8266)

12. HEPES (Sigma-Aldrich, catalog number: H3375)

13. KCl (Sigma-Aldrich, catalog number: S-7653)

14. Sodium pyruvate (Sigma-Aldrich, catalog number: P-4562) (4 °C)

15. CaCl_2_·2H_2_O (Sigma-Aldrich, catalog number: C-7902)

16. KH_2_PO_4_ (Sigma-Aldrich, catalog number: P-5655)

17. MgSO_4_·7H_2_O (Sigma-Aldrich, catalog number: 63138)

18. Glucose (Sigma-Aldrich, catalog number: G-6152)

19. NaOH (Biopack, catalog number: 1641.08)

20. Dimethyl-sulfoxide (DMSO) (Sigma-Aldrich, catalog number: D2650)

21. Dithiothreitol (DTT) (BioBasic, catalog number: DB0058) (-20 °C)

22. NaHCO_3_ (Sigma-Aldrich, catalog number: S6014)

23. 90% Glycerol (Cicarelli, catalog number: 708110)

24. Bromophenol blue (Sigma, catalog number: B-5525)

25. Tween-20 (Sigma-Aldrich, catalog number: SLCL7671)

26. Agarose LE (Productos Bio-Lógicos PB-L, catalog number: IB0501)

27. Source of PKA: recombinant PKA-C (Addgene plasmid, catalog number: 14921, expressed in *E. coli* BL21 and purified with Ni-NTA resin) or sperm extract (detailed here)

**Solutions**

1. H-TYH (non-capacitating media) (see Recipes)

2. CAP 2× (see Recipes)

3. 1.5× Triton lysis buffer (incomplete) (see Recipes)

4. 1.5× Triton lysis buffer (complete) (see Recipes)

5. 5× kinase reaction buffer (incomplete) (see Recipes)

6. 5× kinase reaction buffer (complete) (see Recipes)

7. 2.5× FITC-kinase reaction buffer (see Recipes)

8. Agarose loading buffer 10× (see Recipes)

9. Agarose running buffer 1× (see Recipes)

10. 1% (w/v) agarose LE (see Recipes)

**Recipes**

**1. H-TYH (non-capacitating media)**

**Table:** 

Reagent	Final concentration (mM)	Quantity for 25 mL (mg)
NaCl	119.3	174.3
KCl	4.7	8.8
CaCl_2_	1.71	6.3
KH_2_PO_4_	1.2	4.1
MgSO_4_	1.2	7.4
HEPES (pH 7.2–7.4)	20	119.2
Glucose	5.4	24.8
Sodium pyruvate	0.8	2.2

Dissolve all reagents in Mili-Q water (except for glucose and sodium pyruvate) to a final volume of 25 mL. Sterilize by filtration using a 0.45 μM filter and store at 4 °C for up to 1 month. On the day of the experiment, add 2.2 mg of sodium pyruvate and 24.8 mg of glucose. Then, set aside the volume needed to prepare the capacitating medium, depending on experimental conditions (see Recipe 2). Bring the remaining non-capacitating medium to a final pH of 7.2–7.4 with freshly prepared 5 M NaOH.

**2. CAP 2**×

**Table:** 

**Reagent**	**2**× **concentration**	**Quantity for 5 mL**
NaHCO_3_	40 mM	16.8 mg
BSA	10 mg/mL	50 mg
H-TYH media (Recipe 1)	-	5 mL

Add NaHCO_3_ and BSA to the H-TYH media set aside before adjusting the pH to make CAP 2×. Adjust the CAP 2× pH to 7.2–7.4 right before using, as the pH rises with time. To prepare sperm capacitating condition, add one part of CAP 2× to one part of cells + H-TYH.

**3. 1.5× Triton lysis buffer (incomplete)**

**Table:** 

Reagent	Final concentration	Quantity for 5 mL (μL)
1 M Tris (pH 7.4)	75 mM	465.8
3 M NaCl	225 mM	465.8
100% Triton X-100	1.5%	93.1
Distilled water (_d_H_2_O)		3975.3

Mix Tris, NaCl, and Triton X-100 and aliquot in volumes of 402.5 μL. Store aliquots at -20 °C. One aliquot is enough to lysate approximately five cell pellets.

**4. 1.5× Triton lysis buffer (complete)**

**Table:** 

Reagent	Final concentration	Quantity for 500 μL (µL)
50× protease inhibitor cocktail ROCHE	1.5×	15
10× PhosSTOP ROCHE phosphatase inhibitor	1.5×	75
1 M DTT	15 mM	7.5
1.5× Triton lysis buffer (incomplete) (Recipe 3)	-	402.5

To one aliquot of incomplete 1.5× Triton lysis buffer, add protease inhibitor cocktail, PhosSTOP, and DTT, and keep on ice. DTT is stable for up to 3 years when refrigerated at 2–8 °C. DTT is hygroscopic, has sensitivity to heat, is incompatible with strong oxidizing agents, and decomposes at pH >7.

It is mandatory to use the EDTA-free version of the EDTA-free protease inhibitor cocktail ROCHE 50× because if a chelating agent is present in the kinase reaction medium, it will interfere with the reaction.

PhosSTOP (phosphatase inhibitor cocktail) can be replaced with 15 mM NaF and 100 mM sodium orthovanadate.

**5. 5× kinase reaction buffer (incomplete)**

**Table:** 

Reagent	Final 5× concentration (mM)	Quantity for 3 mL (µL)
1 M Tris (pH 7.4)	250	1363.6
1 M MgCl_2_	50	272.2
_d_H_2_O	-	1363.6

Mix Tris pH 7.4 and MgCl_2_ and bring to a final volume of 3 mL, then separate into 27.5 μL aliquots and store at -20 °C.

Magnesium acts as a mandatory cofactor for PKA catalysis. On the day of the experiment, each aliquot will be supplemented up to a final volume of 50 μL (allowing for 9–10 reaction tubes).

**6. 5× kinase reaction buffer (complete)**

**Table:** 

Reagents	Final 5× concentration	Quantity for 50 μL (µL)
10 mM ATP (_d_H_2_O)	1 mM	5
1 M DTT	50 mM	2.5
50× protease inhibitor Cocktail ROCHE	5×	5
25× PhosSTOP ROCHE	5×	10
5× kinase reaction buffer (incomplete) (Recipe 5)	5×	27.5

To one aliquot of incomplete 5× kinase reaction buffer, add ATP, protease inhibitor cocktail, PhosSTOP, and DTT. Keep on ice.

**7. 2.5× FITC-kinase reaction buffer**

**Table:** 

Reagents	Final Concentration	Quantity for 100 μL (µL)
5× kinase reaction buffer (complete) (Recipe 6)		50
1 mg/mL Kemptide-FITC	7.75 pmol/μL*	10
3.9 mg/mL Kemptide	7.75 pmol/μL*	1.5
_d_H_2_O	-	38.5

To a complete 5× kinase reaction buffer aliquot, add Kemptide and Kemptide-FITC. Keep on ice and protected from light to avoid quenching of the fluorophore.

*Note that 1 µg of Kemptide-FITC + 1 µg of Kemptide (not fluorophore-tagged) will be added per reaction tube. 1 µg of either version of the Kemptide is equivalent to 0.775 nmol.

**8. Agarose loading buffer 10×**

**Table:** 

Reagents	Final 10× concentration	Quantity for 1 mL (µL)
85%–89% Glycerol	63%–66% (v/v)	740
5% bromophenol blue (_d_H_2_O)	0.8% (w/v)	160
100% Tween-20	5% (v/v)	50
_d_H_2_O	-	50

Mix glycerol, bromophenol blue, and Tween-20 and complete to a final volume of 1 mL with _d_H2O. Aliquot and store at -20 °C.

**9. Agarose running buffer 1×**

**Table:** 

Reagents	Final concentration	Quantity for 1 L (mL)
1.5 M Tris	50 mM	33
_d_H_2_O	-	967

Adjust pH to 10.0 with 5 M NaOH before completing the volume with water.

This buffer will be used both to run the electrophoresis and to dilute the agarose, so prepare at least 300 mL of running buffer (250 mL will be needed to fill the electrophoresis tank and 50 mL to prepare a small agarose gel of 7 × 10 cm).

**10. 1% (w/v) agarose LE**

**Table:** 

Reagents	Final concentration	Quantity for 100 mL
Agarose	1% (w/v)	1 g
Tris-HCl 50 mM, pH 10.0	-	100 mL

Heat the solution until the agarose is completely dissolved. Allow the mixture to cool to approximately 60 °C before pouring the solution into the gel tray. Add the adequate gel comb according to the sample number. Prepare on the day of use.

**Laboratory supplies**

1. 0.45 μM filter (Jet Biofil, catalog number: FCA406013)

2. Sterile syringes (Neojet, catalog number: 12238)

3. 15 mL conical tubes (Tarson, catalog number: 546121-RK)

4. 50 mL conical tubes (Tarson, catalog number: 546041-RK)

5. 10 μL pipette tips (Tarson, catalog number: 521000)

6. 200 μL pipette tips (Tarson, catalog number: 521010Y)

7. 1,000 μL pipette tips (Tarson, catalog number: 521016B)

8. 2 mL Eppendorf tubes (Tarson, catalog number: 500020N)

9. 1.5 mL Eppendorf Tubes (Tarson, catalog number: 500010N)

10. 0.6 mL Eppendorf Tubes (Tarson, catalog number: 500000N)

## Equipment

1. Neubauer counting chamber (Marienfeld, catalog number: 0640010)

2. Surgery scissors (Merck, catalog number: Z265977)

3. Forceps (Merck, catalog number: F4142)

4. Vortex mixer (DLab, catalog number: 8031102000)

5. Centrifuge (Eppendorf MiniSpin, catalog number: 5452000010 and Eppendorf 5418 R, catalog number: 5401000013)

6. Water bath (37 °C) (Vicking, catalog number: 1002)

7. Heating block (100 °C) (ThermoFisher, catalog number: 88871001)

8. Agarose electrophoresis equipment (Bio-Rad gel casting system, catalog number: 1704481; Bio-Rad Basic Power supply, catalog number: 1645050)

9. Freezer (-20 °C)

10. Refrigerator (2–8 °C)

11. Micropipettes (Gilson; P2, catalog number: F144054M, P10, catalog number: 144055M, P20, catalog number: F144056M, P200, catalog number: F144058M, P1000, catalog number: F144059M)

## Software and datasets

1. Microsoft^®^ Office Excel (Microsoft Office 365), or similar

2. FIJI (Fiji is just ImageJ) (Open source, imagej.net, 2.16.0)

3. Typhoon FLA 7000 software under the Fluorescence mode or equivalent fluorescence imager

## Procedure

**A. PKA from total sperm extracts (to quantify endogenous PKA activity)**

*Note: During sperm capacitation, there is an increase in cAMP, which binds to PKA-R subunits, triggering the release of the active catalytic subunits. This dissociated and catalytically active enzyme, present in the lysed sperm extracts, is responsible for the posterior phosphorylation of the Kemptide substrate during the kinase assay.*

1. Prepare 1.5× Triton lysis buffer. Add protease inhibitor cocktail and keep it refrigerated (see Recipes).

2. Place two epididymis from sexually matured C57 mice in 600 μL of H-TYH non-capacitating medium and perform swim-out to collect sperm cells (to see how this procedure is performed, refer to Stival et al.) [17].

3. Determine cell concentration using a Neubauer chamber.

4. Calculate the number of total cells present in the final volume of swim-out left. **This data will be used in step A9** to calculate the volume of lysis buffer that will be added to the cell pellet to achieve a suggested concentration of 30 × 10^6^ sperm/mL for the lysis step.

*Note: During step B3, 10 μL of the cell lysate from each experimental treatment will be added to the kinase reaction tubes. This volume should correspond to the lysate obtained from approximately 300 × 10^3^ sperm cells. However, sperm extracts from 50, 100, 200, or 300 × 10^3^ sperm cells in final kinase reaction volumes of 25 μL can also be used [1].*

5. Incubate sperm cells in the conditions to be tested (e.g., *non-cap* and *cap* media). Suggested proportion: 3 × 10^6^ cells/400 μL.

6. Centrifuge sperm at 10,000× *g* for 3 min at room temperature.

7. Carefully discard 370 μL of supernatant (i.e., 93% of the supernatant) so that exactly 30 μL of supernatant is left in the tube.

*Note: If a volume other than 400 μL is used to incubate the cells, adjust the volume to pipette out so that there is always 30 μL of supernatant left in the tube.*

**Warning:** In this step, it is crucial to extract the exact same amount of supernatant from all tubes to compare PKA activity between them. If this step is not done carefully, differences in PKA apparent activity may arise from technical performance that resulted in different dilution factors of the sperm extract.

8. Resuspend the 3 × 10^6^ cell pellet in cold Triton lysis buffer 1.5× to achieve a final volume of 100 μL (note that there should already be 30 μL of supernatant from step A8) and a concentration equivalent to 30 × 10^6^ sperm/mL. So, if 3 × 10^6^ sperm was used in each experimental condition, then add 70 μL of lysis buffer. The final 1× concentration of the **lysis buffer** will be 75 mM Tris-HCl, 150 mM NaCl, EDTA-free protease inhibitor mixture 1×, PhosSTOP cocktail inhibitor 1×, 1% Triton X-100, and DTT 10 mM, pH 7.4.

*Note: If a different volume of sperm cells was used in the previous steps, adjust the volume of lysis buffer so that the final concentration is equivalent to 30 × 10^6^ sperm/mL.*

9. Incubate for 30 min on ice, pipetting up and down every 5 min, making sure not to generate any foam. During this step, start with steps B1 and B2.

10. Sperm extracts are ready to use. Place them on ice until use. From now on, this fraction is named *sperm PKA*.

**B. Prepare reagents needed for kinase reaction**

*Note: During sperm cell lysis, make sure to supplement and prepare all the reagents needed for the kinase reaction (i.e., kinase buffer 5×, Kemptide, Kemptide-FITC, and loading buffer 10×). Also, prepare agarose 0.8%–1.5% gel and running buffer (see Recipes).*

1. Supplement 5× kinase buffer: On the day of the experiment, add to each 27.5 μL aliquot of 5× kinase reaction buffer (incomplete) the ATP, DTT, protease inhibitor, and PhosSTOP to prepare 5× kinase reaction buffer (complete, see Recipe 6).

2. Prepare 2.5× FITC reaction buffer: to the 5× kinase reaction buffer, add Kemptide and FITC-Kemptide to prepare 2.5× FITC reaction buffer (see Recipe 7). This volume is enough for 9 or 10 reactions (keep on ice and protected from light).

3. Prepare reaction tubes as follows, while **keeping them on ice:**

**Table:** 

Reagent	Volume	
Sample (PKA)*	1–15 μL	
_d_H_2_O**	*qs* 25 μL
2.5× FITC kinase reaction mix	10 μL

*“Sample” can either refer to recombinant PKA-C, partially purified PKA, or sperm extracts to assess PKA activity.

**cAMP might be added to serve as a positive control for Kemptide phosphorylation when the sperm extract is used as a source of PKA. In this case, add 5 μL of 5 μM cAMP to achieve a final concentration of 1 μM.

*Notes:*

*1. The volume of Kemptide and Kemptide-FITC added to the reaction tube is equimolar to achieve a final total substrate concentration of 62 μM. The stock concentrations are thought to add 1 µg of each substrate (775 pmol = 31 μM for each substrate); MW Kemptide = 772 g/mol and MW KemptideLys8-FITC = 1289.5 g/mol.*

*2. Consider including positive and negative phosphorylation controls. The* ***positive control*** *can be made by either (1) replacing the cell lysate with active recombinant PKA-C or (2) adding 1 μM cAMP to the sperm lysate. The* ***negative control*** *can be made by replacing the cell lysate with either (1) cell-free lysis buffer, (2) recombinant PKA-C dilution buffer, or (3) distilled water [1].*

*3. Keep all tubes with KempLys8-FITC in the dark at all times.*

*4. Start the kinase reaction by adding the required amount of sample/PKA to each tube.*

4. Incubate for 25 min at 37 °C in the dark.

5. Place the tubes on ice to slow down the reaction and add 2.8 μL of loading buffer 10 × [glycerol 65% (v/v) + bromophenol blue 0.8% (w/v) + Tween-20 5%] to each tube.

6. Incubate tubes for 60 s at 100 °C.

7. Centrifuge at 10,000× *g* for 1 min at 4 °C.

8. Transfer 20 μL of supernatant to a new tube.

9. Store at -20 or -80 °C, protected from light, or run the gen immediately.

**C. Run agarose electrophoresis**

1. Prepare running buffer for electrophoresis. Note that this buffer will be used both to run the electrophoresis and to dilute the agarose.

*Note: Prepare at least 300 mL of running buffer (250 mL will be needed to fill the cube and 50 mL to prepare a small agarose gel of 7 × 10 cm).*

2. Prepare a solution of 1% (w/v) of agarose LE diluted in 50 mM Tris-HCl (pH 10.0) buffer. To completely dissolve the agarose powder, microwave the solution at 30% power.

3. Assemble the horizontal agarose gel apparatus following the manufacturer’s instructions.

*Notes:*

*1. As a general reference, for a 7 × 10 cm gel, approximately 30 mL of agarose solution is needed, while for a 10 × 15 cm gel, approximately 70 mL of agarose solution is needed.*

*2. As a general suggestion, a smaller and thinner gel is better since the bands will be less diffuse.*

*3. If the gel will not be used within 40 min after its polymerization, cover it with a thin layer of agarose running buffer and store at room temperature for a maximum of 2 h.*

4. Heat the solution until the agarose is completely dissolved (microwave at 30% power until completely dissolved). Allow the mixture to cool to approximately 60 °C before pouring the solution into the gel tray.

5. Place the comb at the top of the gel tray. If more than one comb is used, arrange them with at least 3 cm of gel space between the combs.

6. Allow the gel to solidify for about 20 min until it turns opaque.

7. Gently pull the comb from the solidified 1% (w/v) agarose gel. Place the gel tray in the electrophoresis tank and pour agarose running buffer over the gel until the wells are filled and the buffer covers the gel completely. Protection from light is not strictly necessary.

8. Load the same volume of sample in each well. This step might need setting depending on the specific requirements of the available imager to avoid under-detection or saturated signals. Suggested amount: 0.4 µg of Kemp-FITC (4 μL).

*Note: To minimize diffusion and blurred fluorescence, load the samples as quickly as possible without overflowing the lanes. Leave a free well between samples if possible.*

9. Immediately after the last sample is loaded, run the gel at 80–100 V until the running front runs out of the gel (approximately 45 min). Protection from light is not required for standard 30–50 min electrophoresis runs. However, we recommend minimizing exposure to intense ambient lights.

*Note: High salt concentrations in the samples may retard the mobility of the peptide. In such cases, the gels should be run slightly longer to ensure that all products have completely left the wells.*

10. When electrophoresis is complete, remove the gel from the chamber and place it in a container covered with running buffer to prevent dehydration of the gel.

**D. Image acquisition**

**Figure 1.**
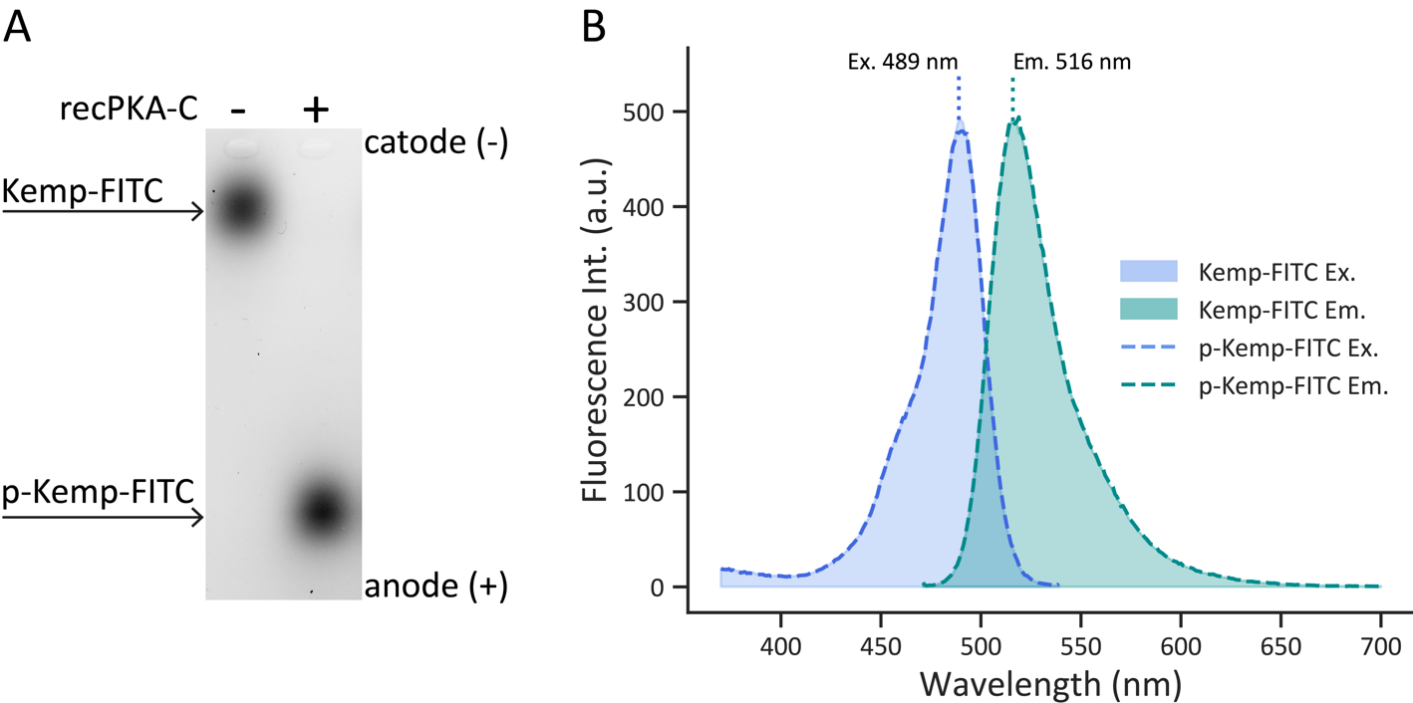
Comparison of non-phosphorylated vs. phosphorylated Kemptide-FITC spectra. (A) Representative agarose gel electrophoresis image obtained after KiMSA. The left lane contains non-phosphorylated Kemptide-FITC, and the right lane contains Kemptide-FITC incubated with recPKA-C at 37 °C for 30 min. (B) Excitation (blue, left, λem = 550 nm) and Emission (green, right, λex = 460 nm) spectra of Kemptide-FITC (shaded area) and phosphorylated Kemptide-FITC (p-Kemp-FITC) (dashed lines). No spectral shifts were observed between phosphorylated and non-phosphorylated Kemptide-FITC. Excitation and emission maxima are 489 nm and 516 nm, respectively.

The excitation and emission spectra of non-phosphorylated and phosphorylated Kemptide-FITC were obtained to assess whether phosphorylation induced a spectral shift. Kinase reactions were prepared with Kemptide-FITC either in the presence or absence of 1.5 μg/mL (7.5 mU/mL) recPKA-C, in order to generate fully phosphorylated (100%) and non-phosphorylated (0%) Kemptide-FITC, respectively (Figure 1A). Reactions were incubated at 37 °C for 30 min.

Spectral scans were performed in the range of λ = 450 nm to λ = 700 nm, with excitation set at λ = 473 nm for emission measurement and emission set at λ = 520 nm for excitation measurements. Prior to sample analysis, the excitation and emission spectra of water were recorded to correct the FITC spectra (Figure 1B). The excitation and emission spectra do not present significant shifts between the phosphorylated and non-phosphorylated forms of Kemptide-FITC. This allows, when analyzing agarose gels from KiMSA experiments, the quantification by densitometry of the phosphorylated and non-phosphorylated forms of Kemptide-FITC, without the need to introduce mathematical corrections.

In order to acquire the image from the agarose gel after electrophoresis, do the following:

1. Configure the gel imager (e.g. Typhoon) to acquire fluorescence signal:

Excitation 490 nm

Emission 520 nm

*Note: High emission of FITC might saturate image acquisition. Setting to higher emission wavelengths for detection (570–590 nm) might prove useful (Figure 1A).*

2. Acquire image, adjusting photomultiplier tube (PMT) sensitivity accordingly (Figure 1B).

3. Export as TIFF to analyze band densitometry with Fiji software.

## Data analysis

**A. Calculation of enzymatic activity**

Scanned images of gel electrophoresis were analyzed with Fiji to quantify band densitometries, as previously explained [20].

1. Select a vertical region of interest (ROI) covering each lane.

2. Calculate densitometry signals (absorbance) for all of the Kemp-FITC and pKemp-FITC signals in each condition.

*Note: If some pKemp-FITC signal was detected in the negative control (i.e., Kemptide-FITC without PKA), the densitometry corresponding to that band should be subtracted from all the other conditions.*

3. Calculate the relationship between the total FITC signal (i.e., the sum of densitometries for Kemp-FITC and p-Kemp-FITC in each lane) with the total amount of peptide loaded in the gel. As a direct reference for total FITC signal, use the densitometry calculated for the negative control. To establish the relationship between the detected fluorescence and the amount of peptide, consider the volume of the kinase reaction loaded in each lane. For example, if 0.78 nmol of Kemp-FITC was added to the 25 μL kinase reaction, loading 4 μL into the gel would correspond to having 0.1248 nmol of FITC in each gel lane.

4. Determine the amount of nmol of phosphorylated Kemptide-FITC (pKemp-FITC) in the gel for each condition.

$$pKempFITC(nmol)=pKempFITC(densitometry)\frac{total\;KempFITC(nmol)}{total\;KempFITC(densitometry)}$$

5. Calculate the **total** amount of product generated during the kinase reaction, including both non-labeled and FITC-labeled phospho-Kemptide. To do this, adjust the nmol of p-Kemp-FITC determined in step 4 by applying the appropriate dilution factor, considering the volume loaded in gel relative to the total volume of the kinase reaction (e.g., if the kinase reaction was done in 25 μL and then only 4 μL were loaded in the gel, the dilution factor would be 25/4 = 6.25). Then, account for the fact that equal amounts of non-labeled Kemptide and FITC-labeled Kemptide were initially added as substrates, meaning that equal amounts of non-labeled and labeled phosphorylated products were formed.

$$reaction\;pKemp\left(nmol\right)=2\times pKempFITC(nmol)\frac{Reaction\;volume}{Loaded\;volume}$$

6. Calculate PKA enzymatic activity by estimating the number of international units (IU), defined as the amount of enzyme that catalyzes the conversion of 1 μmol of substrate into product per minute under specified conditions. For kinases, this means that 1 IU is equivalent to the amount of enzyme that transfers 1 μmol of phosphate to its substrate per minute.

Note that since only one phosphorylation site exists on each Kemptide, the moles of Kemptide calculated in step 5 are equivalent to the number of moles of phosphate transferred from ATP.

To determine the number of international enzymatic units (IU), first divide the value of transferred phosphates μmol in the reaction (calculated in step 5) by the reaction time (25 min).

IU (µmol/min)=reaction pKemp (nmol)103reaction time

**General note on the definition of enzymatic unit:** 1 unit (IU) is the amount of enzyme that catalyzes the reaction of 1 μmol of substrate per minute (definition A).

In most R&D settings, 1 μmol of substrate represents a relatively large amount, rendering it an impractical unit, and other definitions may be preferred. The following non-standard definition is commonly used:

1 unit (IU) is the amount of enzyme that catalyzes the reaction of 1 nmol of substrate per minute (definition B).

Note that the change in definition has a profound effect on the stated number of units, i.e., 1 unit of enzyme according to definition A would equate to 1000 units according to definition B!

Specific enzyme activity (usually stated simply as *specific activity*) is the number of enzyme units per milliliter divided by the concentration of protein in mg/mL. Specific activity values are therefore quoted as units/mg or nmol/min/mg (if unit definition B is applied).

Specific activity is an important measure of enzyme purity, and values for different batches of a pure enzyme should be the same, within normal experimental error.

Serial dilutions of an enzyme solution will have different enzyme activity values but identical specific activity values, because while calculating specific activity, the numerator (units/mL) and denominator (mg/mL) are affected equally by sample dilution.

To calculate activity normalized by sperm cell number:

$$Activity\;(\frac{nmol}{1\;Msperm\times min})=\frac{reaction\;pKemp\left(nmol\right)\times \frac{sperm\;cells}{10^{6}}}{reaction\;time}$$

## Validation of protocol

This protocol has been used and validated in the following research article:

Novero et al. [1]. A versatile kinase mobility shift assay (KiMSA) for PKA analysis and cyclic AMP detection in sperm physiology (and beyond). *Front Cell Dev Biol* (Figures 3A and 4A).

## General notes and troubleshooting

**General notes**

1. Precision in volume measurements and calculations is crucial, as even minor discrepancies can introduce errors in FITC densitometry, potentially leading to incorrect attribution of phosphorylation changes to PKA activity.

2. The FITC-Kemptide substrate is sensitive to light and freeze-thaw cycles. Store in small aliquots at -20 °C, protected from light. Avoid repeated freeze-thaws, and thaw on ice immediately before use.

3. Always include both positive (recPKA-C or 5 mM ATP) and negative (no ATP or lysis buffer without cells) controls.

4. For cell lysates, ensure uniform lysis conditions across samples.

**Troubleshooting**

Problem 1: One or more conditions reach 100% PKA activity, possibly hiding a difference in activity between samples.

Possible cause: Kemp-FITC substrate was fully phosphorylated within the kinase reaction time, reaching a reaction plateau.

Solution: Reduce kinase reaction time or temperature, so that all conditions show under 100% kinase activity.

Problem 2: Weak or no fluorescent signal on the agarose gel.

Possible cause: Insufficient FITC-Kemptide concentration.

Solution 1: Confirm peptide stock is correctly diluted and stored; adjust kinase reaction concentration as needed.

Possible cause 2: Fluorophore photobleached or degraded.

Solution 2: Protect FITC-Kemptide from light. Store at -20 °C in aliquots. Do not expose aliquots to more than two freeze-thaw cycles.

Possible cause 3: Incorrect imager excitation/emission settings.

Solution 3: Ensure gel imager or scanner is configured for FITC (excitation ~488 nm, emission ~520 nm) and that gain/sensitivity settings are adequate.

Problem 3: No clear mobility shift between phosphorylated and unphosphorylated peptides.

Possible cause 1: Agarose gel percentage is too low or too high.

Solution 1: Optimize agarose concentration (usually 1.5%–2% w/v) to improve resolution of small mobility shifts.

Possible cause 2: Migration time is too short.

Solution 2: Allow electrophoresis to run longer to increase separation.

Possible cause 3: Wrong running buffer pH.

Solution 3: Check the running buffer pH is around 10 to ensure a correct FITC-Kemptide protonation state required for mobility differences.

Problem 4: High background or multiple bands.

Possible cause 1: Nonspecific kinase activity in lysate.

Solution 1: Include specific PKA inhibitors (e.g., PKI) in controls to confirm specificity.

Possible cause 2: Sample degradation.

Solution 2: Add protease inhibitor to the sample before initial centrifugation (before step A6).

Possible cause 3: Contaminants from sperm lysate.

Solution 3: Include proper negative controls.

## Supplementary information

The following supporting information can be downloaded here:

1. Table S1. PKA activity calculation sample sheet.
